# Assessing the quality of drinking water from selected water sources in Mbarara city, South-western Uganda

**DOI:** 10.1371/journal.pone.0297794

**Published:** 2024-03-28

**Authors:** Catherine N. Abaasa, Savino Ayesiga, Julius B. Lejju, Morgan Andama, Imelda K. Tamwesigire, Joel Bazira, Frederick Byarugaba

**Affiliations:** 1 Faculty of Medicine, Mbarara University of Science and Technology, Mbarara, Uganda; 2 Faculty of Science, Mbarara University of Science and Technology, Mbarara, Uganda; 3 Faculty of Science, Muni University, Arua, Uganda; University of Cape Coast, GHANA

## Abstract

This study assessed the physical, chemical, and microbiological quality with emphasis on risk score, source apportionment, geochemistry, feacal coliforms and water quality index of drinking water from selected water sources. A cross-sectional study was conducted in six villages in Mbarara city, south-western Uganda. Each selected source was inspected using a WHO-adopted sanitary inspection questionnaire. Each source’s risk score was calculated. Thirty-seven samples were taken from one borehole, nine open dug wells, four rain harvest tanks, and twenty-three taps. The values for apparent color and phosphate were higher than the permissible level as set by the World Health Organization and Ugandan standards (US EAS 12). The isolated organisms were *Klebsiella spp*. (8.11%), *Citrobacter divergens* (62.16%), *Citrobacter fluendii* (2.7%), *E*. *coli* (35.14%), *Enterobacter aerogenes* (8.11%), *Enterobacter agglomerus* (5.4%), *Proteus spp*. (2.7%), *Enterobacter cloacae* (13.5%), and *Proteus mirabilis* (2.7%). Twelve water sources (32.4%) had water that was unfit for human consumption that was unfit for human consumption (Grade E), Five sources (13.5%) had water that had a very poor index (Grade D), nine (24.3%) had water of poor index (Grade C), eight (21.6%) had water of good water index (Grade B), and only three (8.1%) had water of excellent water quality index (Grade A). The piper trilinear revealed that the dominant water type of the area were Mgso_4_ and Caso_4_ type. Gibbs plot represents precipitation dominance. PCA for source apportionment showed that well, tap and borehole water account for the highest variations in the quality of drinking water. These results suggest that drinking water from sources in Mbarara city is not suitable for direct human consumption without treatment. We recommend necessary improvements in water treatment, distribution, and maintenance of all the available water sources in Mbarara City, South Western Uganda.

## Introduction

As a fundamental human right, quality and safe drinking water should be accessible, adequate in amount, free of pollution from any harmful microorganisms and chemicals, safe, and easily available all year round [1]. Unsafe and low-quality water or a lack of access to water impacts people’s livelihoods, including their dignity and socioeconomic growth [2]. United Nations Sustainable Development Goal 6 aims at achieving access to a clean, safe, and high-quality water supply by communities to reduce the danger of outbreaks caused by water-borne infections hence promoting economic growth [3]. It should be noted that; groundwater accounts for approximately 30.1% of the freshwater that is at a high risk of contamination from anthropogenic activities and climate change effects [4]. The physical, chemical and microbiological quality of drinking water depend on the geological formation of the area [5]. Equally unregulated community members’ practices lead to pollution of water resources since water is contaminated by undesired substances both from natural sources (mining, industrialization, waste disposal, and urbanization) and agricultural operations [6, 7].

The World Health Organization reported that, between 2000 and 2020, 1 in 4 people around the world lacked safely managed drinking water and continued to rely on unimproved water sources like unprotected wells, springs, and surface water, with nearly half of those people using unimproved drinking water from sub-Saharan Africa and 2 out of 5 people still lacking safely managed sanitation [8]. Approximately 38 million people (83% of the population) in Uganda lack access to a reliable, safely managed source of water, and 7 million people (17%) lack access to improved sanitation solutions [9]. Mbarara city is a newly created city in Uganda that is faced with challenges of informal and unplanned settlements amidst scarce social services. River Rwizi the main source of water supply for treatment and distribution in the city is frequently contaminated by the careless discharge of sewage waste from factories, metal workshops, and other human activities, which alters its physical-chemical properties and microbiological quality [10]. Human activities such as sand mining, brick manufacturing, farming, and watering animals in midstream have all caused the river to deteriorate. Water hyacinth has choked it, causing floods and silting. Mudslides often result due to environmental deterioration brought about by bush burning and agricultural practices in the River Rwizi’s upstream catchment zones [11]. The increase in population in Mbarara city has resulted into poor sanitation practices amidst low toilet coverage and substandard solid waste disposal management [12]. Leakage into wells or boreholes, rivers and broken water distribution pipes leads to adverse effects like development of antibiotic-resistant bacteria, ecotoxicological effects, and several endocrine disorders [13]. This study studied the physical, chemical and microbiological quality of open dug wells, Rainharvest tanks, boreholes and piped water from six villages in Mbarara city Southwestern Uganda with the aim to (1)Physical chemical and bacteriological parameters of the selected drinking water sources (2)investigate the geochemistry responsible for the drinking water quality and (3) establish the water quality index of the selected drinking water sources to ascertain their suitability for drinking water use.

## Methods and materials

### Study site

**Fig 1:**
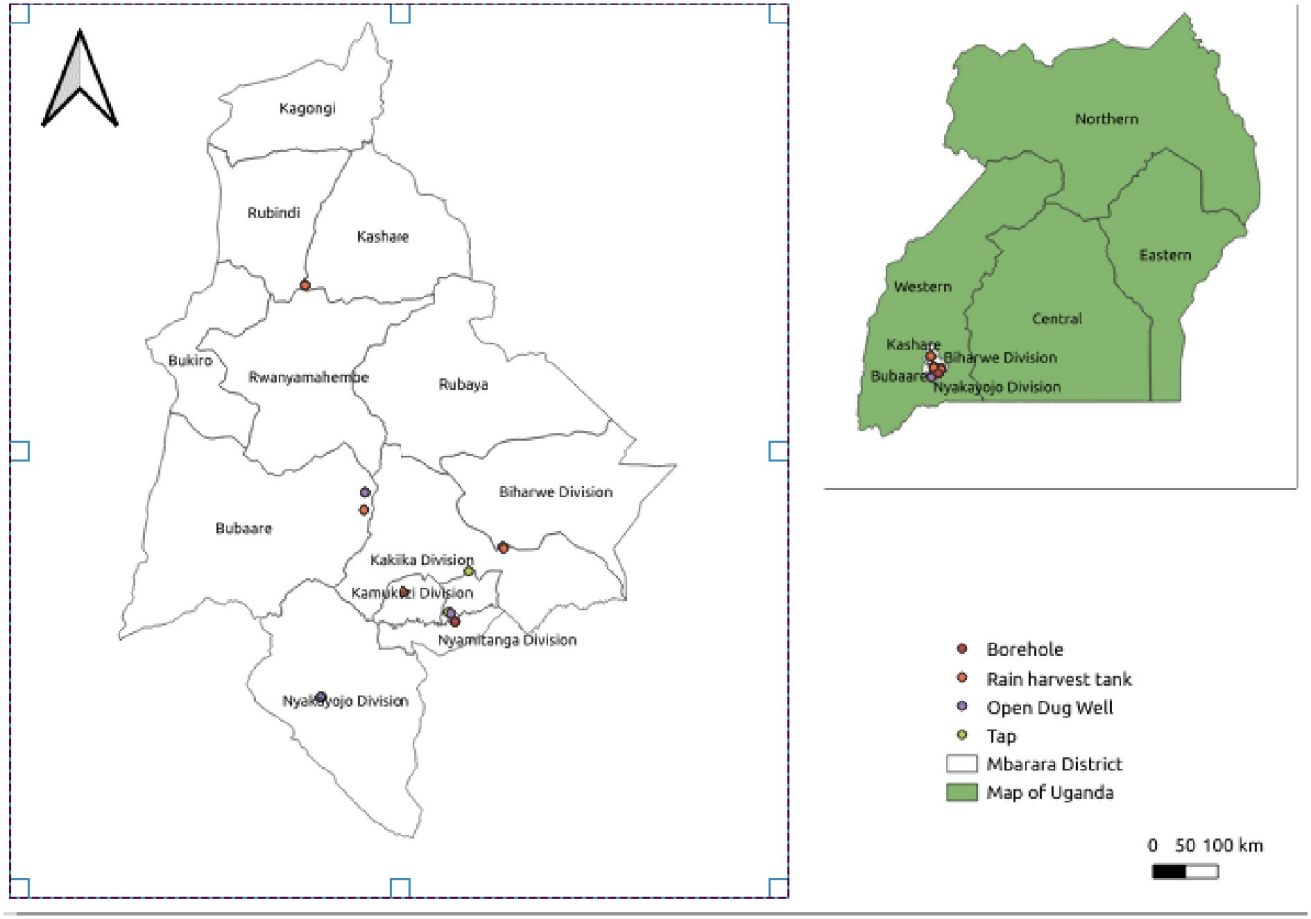
Map of study area in Mbarara city showing the location of the selected drinking water sources (” figure is similar but not identical to the original image and is therefore for illustrative purposes only”).

This study was conducted in Mbarara City, south-western Uganda. Mbarara City is the newly created commercial and administrative capital of Mbarara District in south-western Uganda. Mbarara City is the second-biggest city in Uganda and is faced with increased population growth and increased infrastructure development. Mbarara city is located 270 kilometers by road, southwest of the capital city, Kampala. Mbarara district lies between coordinates 00 36 48 S and 30 39 30 E and covers an area of 1,778.4 square kilometers (Fig 1). It has a population of 91867 [14]. Mbarara city receives an average annual rainfall of 1200 mm, with two rainy seasons during the months of September–December and February–May. Temperature ranges between 17 °C and 30 °C, with a humidity of 80–90%. The topography is a mixture of fairly rolling and sharp hills and mountains, shallow valleys, and flat land. Mbarara City is provided, operated, and maintained with safe water supply technologies and sanitation facilities for all communities in the city. Mbarara district recorded an increase in access to safe and clean water from 45% in 2000 to about 63% in the villages and 65% for the municipality in 2007. Safe water coverage is 65.9% in rural areas and 95.7% in urban areas, while accessibility to safe water lies between 29% and 95% [15].

### Study design and data collection

This was a quantitative cross-sectional study on selected drinking water sources in Mbarara city. Mbarara City has a total of 23 wards spread across six divisions and constituencies. Judgmental sampling was employed [16]. Administrative clearance was obtained from district, city, parish, National Water and Sewerage Cooperation, and the Ministry of Water, Lands, and Environment authorities. The protocol was reviewed and approved by the Mbarara University of Science and Technology Institutional Review Committee (MUST-2021-39) and the National Council of Science and Technology (HS1469ES). Permission was obtained from the district, local council leaders, and household heads, especially for water harvest tanks, before the commencement of data collection. Three divisions of Kakoba, Kakiika, and Nyakayojo were randomly selected. A ward was randomly selected from each of the three selected divisions. From each of the selected parishes (Nyarubanga, Rubiri, Lugazi, Kaburangiire, Katebe, and Katukuru), a village was selected. A total of six villages were selected and surveyed to identify the water sources. The selected communities were mapped, and all the drinking water sources used by them were listed. From each of the listed water sources, approximately 50% were sampled in selected wards and divisions between May and June 2022. However, all the wells, boreholes, and rainwater in each selected village were samples since there were very few. A total of six villages were selected and surveyed to identify the water sources.

### Survey

**Fig 2:**
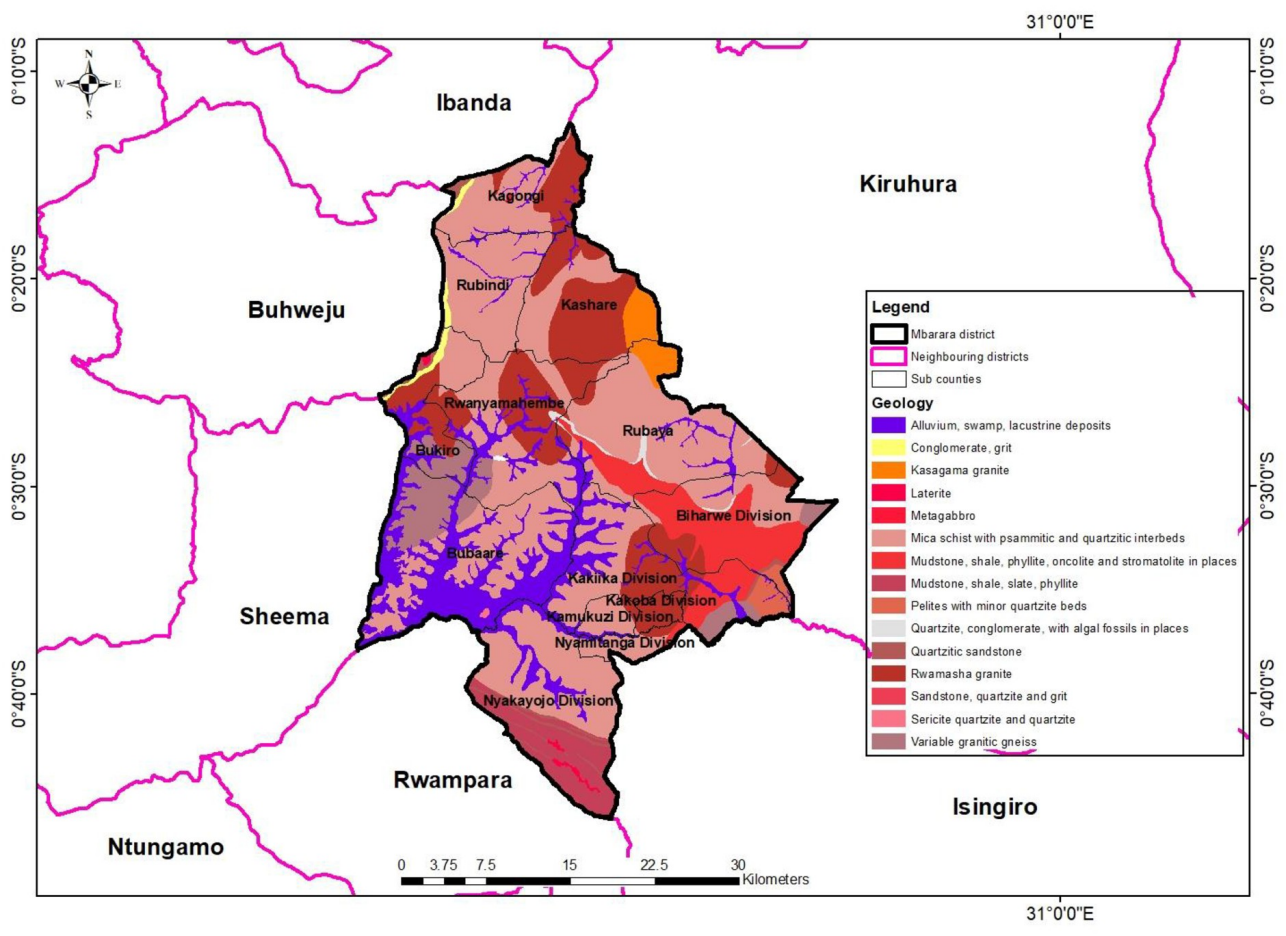
Geographical map of the sampled drinking water sources.

Permission to access the selected villages was sought from one (1) chairperson of the selected villages, who introduced us to the village members. Permission to collect water samples from the community water sources was sought from the water source owners. A sanitary inspection form (adopted from World Health Organization) [2] was used to assess the sanitary conditions around the sampled water sources. The research assistant filled out a WHO sanitary inspection form consisting of a set of questions with” yes” or no” answers for every selected water source. All types of water sources that are used to supply water for human consumption in the selected villages in Mbarara City were included in the study. All water sources that were damaged and nonfunctional were excluded. Geographic Coordinates of drinking water sources were collected using a handheld GPS. The coordinates generated were used to plot the geological map (Fig 2).

### Sample collection

Samples were taken from locations that were representative of the water source. Water samples were randomly collected from sources representing environments ranging from high to low presumptive physical, chemical, and microbiological pollution risk to communities. Samples for analysis were aseptically collected from the selected water sources into sterilized 250-ml glass collection containers. A minimum of two samples (“spot” and “snap”) were collected. The tap nozzles were flamed, the water allowed to run for about 2 minutes, and four samples were collected, two for microbiological testing and two for chemical testing. Boreholes were pumped for up to 15 minutes to purge the aquifers and minimize contamination before sample collection. Tests on the microbiological quality and safety were conducted as per the standard microbiological procedures, following standard operating procedures [17] that were prepared and customized according to the study protocol. 1% sodium thiosulfate was used to neutralize any chlorine in water samples treated with chlorine. The standard operating procedures were diligently adhered to during the study. A total of thirty-eight (38) water sources were selected, and only thirty-seven (37) were sampled from the selected six (6) villages of the six parishes in Mbarara city, south-western Uganda. One selected borehole was found dysfunctional at the time of data collection. The sampled water sources were inspected for their sanitary conditions around the water source. The physical, chemical, and microbiological properties of the water sampled were tested in the months of May and June 2022.

### Sanitary inspection of the selected drinking water sources

The sampled water sources were inspected each with a study-customized specific inspection form adopted from the revised 2018 WHO sanitary inspection forms. The sanitary inspection forms consisted of a set of questions relating to the presence of potential sources and pathways of contamination specific to the different drinking water sources: open dug wells, rainwater harvest tanks, tap water, and boreholes. A risk score was computed based on 9–10 as very high risk, 6–8 as high risk, 3–5 as medium risk, and 0–3 as low risk.

### Physicochemical parameter analysis

**Fig 3:**
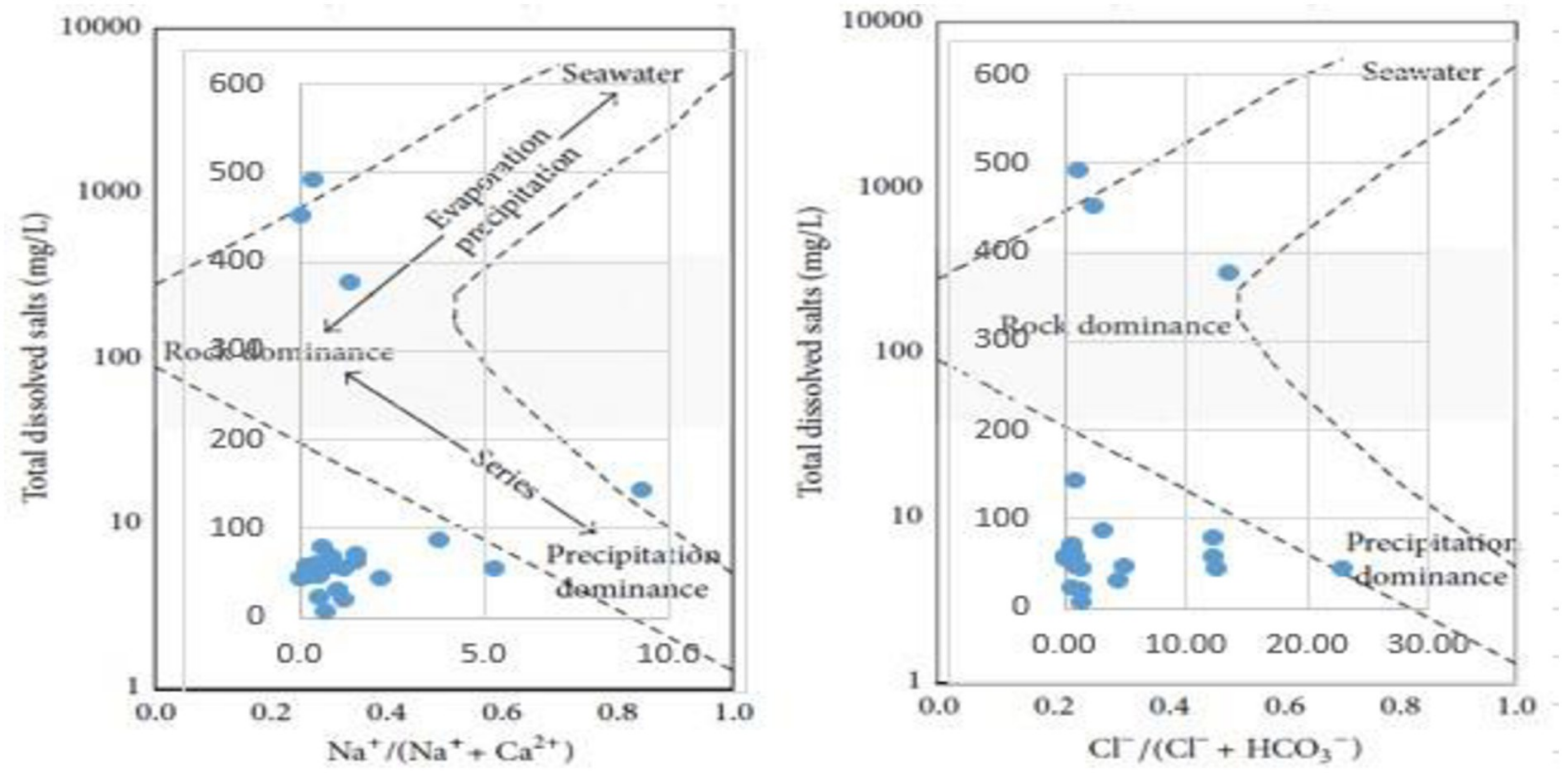
Gibbs plot shows the control mechanism of drinking water chemistry. Drinking water data were plotted as a) Na/Na+Ca Mg/L against Log TDS and b) Cl/Cl+HCO_3_mg/L against Log TDS.

The sampled water was tested for Apparent color, Temperature, Hydrogen potential (pH), Turbidity (turb), Electrical conductivity (EC), Dissolved oxygen (DO), Phosphates (PO_4_), ammonia, Total Suspended Solids (TSS), and Chloride. The physicochemical parameters were determined using a multiparameter meter (**HI-98196 multiparameter waterproof meter**). The instruments were calibrated in accordance with the manufacturer’s guidelines before taking the measurements. The value of each sample was taken after submerging the probe in water and held for a couple of minutes to achieve a reliable reading. After measurement of each sample, the probe was rinsed with deionized water to avoid cross-contamination among different samples. At each site, the water parameters were determined twice, hence two replicates. The apparent color of the water was determined using a photometer **(HI-83303-02**). A volume of 50 ml of water was collected from each water source visited and taken to the Biology Laboratory at Mbarara University of Science and Technology for analysis of the color of the water following standard protocols and methods of the American Public Health Association (APHA) [18]. A Gibbs plot was plotted to show the control mechanism of drinking water chemistry (Fig 3).

### Microbiological water quality analysis

For microbiological analysis, water samples were delivered to the Microbiology Laboratory of the Department of Microbiology of Mbarara University of Science and Technology (MUST) in an ice-cooled box. Using aseptic procedures, water samples were diluted 1 ml each in 9 ml of sterile field phosphate buffer. Approximately 1 ml of each sample was diluted into separate sterile fields of phosphate buffer (9 ml) using a sterile micropipette tip. This was diluted tenfold down to 10^4^. A volume of 1 ml of each of these dilutions was dropped into the middle of a sterile petri dish, and 18 ml of molten cooked eosine methylene blue (Levine) was poured into each petri dish, rocked several times, left to solidify, and incubated at 37°C for up to 48 hours. The colonies that formed were counted depending on their color: metallic sheen with a dark center for *E*. *coli*, brown center for *A*. *aerogenes*, and pink for non-lactose fermenting gram-negative bacteria [19]. The dilutions were cultured in duplicate, and the average was taken. The final counts were reported as colony-forming units per liter (CFU/ml). The blue colonies were subcultured on MacConkey plate and biochemical tests (Triple sugar iron agar, SIM, methyl red and Voges Proskaeur) were performed to identify Enteropathogens [20].

### Quality control and quality assurance

All glassware were thoroughly washed and rinsed with deionized and dried in an oven. Media and all the reagents of analytical grade were purchased from Joint medical stores and they were within their shelf life. Standard operating procedures were prepared by the principal investigator according to manufacturer’s instructions and were adhered to throughout the sample collection, transportation and processing. Research assistants were trained on standard operating procedures before commencement of the study. Instruments were calibrated using standards before quantification and analysis. Quality control checks were conducted as stipulated in the standard operating protocol for every procedure.

### Data management and analysis

Data was entered into a Microsoft Excel data sheet created specifically for the study. It was checked for completeness, and the data was cleaned to ensure consistency. Data on the sanitary conditions of the various water sources was analyzed using the source-specific risk score. Descriptive statistics of isolated microorganisms, chemical, and physical properties were reported. ANOVA and principal component analysis were used to help understand the water quality parameters by village and water source. Hierarchical cluster analysis was performed to understand the constructed groups of the physical chemical observations of drinking water. pH, turbidity, electrical conductivity, total suspended solids, dissolved oxygen, ammonia, phosphorus, chloride, and fecal coliform were the parameters considered to compute the water quality index. The weighted arithmetic method developed by Brown et al. (1972 was used. It is simple to use and interpret the water quality index based on the weighted arithmetic average of individual water quality parameters. The water quality index (WQI) for each water source was computed according to the following formula:

$$\text{Unit}\;\text{weight}\;\text{factor}\left(wn\right)=\frac{K}{Sn}$$ (1)

Where Sn is the standard desirable value of the nth parameter and K is the constant of proportionality.


$$K=\frac{1}{\frac{1}{S1}+\frac{1}{S2}+\frac{1}{S3}+\ldots }=\frac{1}{\sum \frac{1}{Sn}}$$ (2)

The total of all specified parameter unit weights factors *wn* = 1

where

$$QpH\left(\text{pH}\;\text{ideal}\;\text{value}\right)=\frac{\left[Vn-V0\right]}{\text{Sn}-\text{V}0}*100$$ (3)

*Vn* is the average concentration of the nth parameter, Sn is the standard desirable value of the n^th^ parameter. *V*0 is the actual value of the parameter in pure water which in most cases is zero except for P^H^7.0 and dissolved oxygen 14.6mg/l [21].


$$WQI=\frac{\sum WnQn}{\sum Wn}$$ (4)

The following is the interpretation of water quality index (WQI) for the water quality status (Table 1), 0–25 (excellent), 26–50 (good), 51–75 (poor), 76–100 (very poor), >100 (unfit for human consumption) [22].

**Table 1:** Water quality index and grade.

Water quality grading based on the Arithmetic WQI Classification	Status	Grading
0–25	Excellent	A
26–50	Good	B
51–75	Poor	C
76–100	Very poor	D
>100	Unfit for human consumption	E

## Results

Water samples from 37 water sources were collected and tested for physical, chemical, and microbiological safety and quality and their suitability for drinking as per Ugandan and WHO guidelines for drinking water [23]. A water quality index was computed for each source from the measured values of the physical, chemical, and microbiological parameters. A sanitary inspection was conducted for each of the sampled water sources, and the status was ascertained using risk scores as presented below.

### Sanitary inspection of the selected drinking water sources

**Fig 4:**
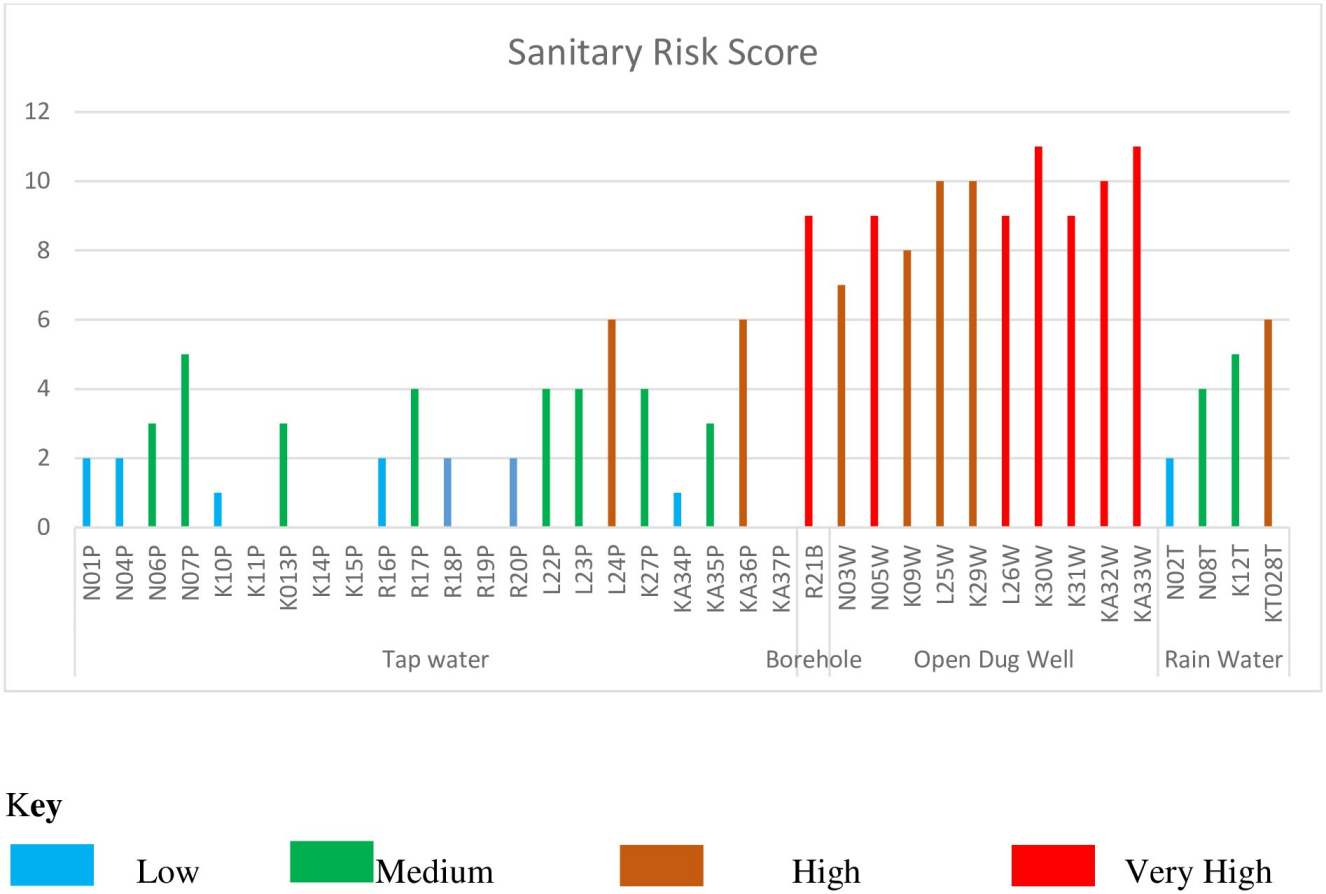
Sanitary inspection of the selected drinking water sources.

Six open dug wells (N05W, L025W, K030W, KA031W, KA32W, and KA33W) and one borehole (R022B) were in environments that were at very high risk. Three (3) taps (L24P, L25P, and KA36P) and one (1) rain harvest tank (KT028T) were in environments that were at high risk. The rest of the water sources were in environments of low to medium risk as shown in Fig 4.

### Physical chemical properties of samples collected from selected drinking water sources

Using the ANOVA test for statistical differences in mean physical and chemical water properties with respect to the village where the water samples were collected, apparent color, temperature, turbidity, electrical conductivity, phosphorus, ammonia, total suspended solids, and chloride were not statistically significant. P^H^ (F-ratio: 34.58, <0.001) and dissolved oxygen (F-ratio: 45.1, <0.001) were statistically different across sites as shown in Table 2.

**Table 2:** Median and interquartile range of physical chemical properties with respect to village.

Village	Kaburangire	Katukuru	Katebe	Lugazi	Nyarubanga	Rubiri	F-Ratio	P-value
**Apparent Color (Median IQR)(TCU)**	8(0–26)	53(36–66)	44(7–114)	5(1–28)	17(13–54)	25(16–42)	1.30	0.2890
**Temperature** °**C**	24(20–25)	23(23–24)	23(22–24)	24(23–25)	25(24–27)	24(23–25)	2.35	0.0637
**PH**	4(4–4)	4(4–4)	4(3–4)	4(4–5)	6(5–6)	4(4–4)	34.58	<0.001
**Turbidity (NTU)**	4(4–9)	4(4–4)	21(2–32)	4(3–20)	3(3–10)	8(8–9)	1.90	0.1223
**Electrical conductivity (μs/cm)**	124(123–142)	115(114–116)	97(63–112)	118(118–122)	122(103–132)	117(116–118)	0.66	0.6592
**Dissolved oxygen (mg/l)**	4(3–4)	0.9(0.9–0.9)	0.9(0.8–0.9)	4(3.6–3.9)	5(4–5)	4(3.6–4)	45.18	<0.001
**Phosphorus (mg/l)**	1(0.8–2)	1(0.7–4)	0.8(0–5.3)	0.4(0.3–0.6)	0.8(0.7–0.9)	0.8(0.4–0.8)	1.62	0.1829
**Ammonia (mg/l)**	0.1(0.1–0.2)	0.04(0.03–0.32)	0.14(0.05–0.24)	0.07(0.05–0.24)	0.5(0.2–0.6)	0.2(0.2–0.3)	0.67	0.6461
**Total suspended Solids(mg/l)**	62(61–71)	57(57–88)	44(32–57)	59(48–59)	61(52–66)	58(58–59)	0.58	0.7128
**Chloride(mg/l)**	0.71(0.67–1.3)	0.7(0.7–1)	0.07(0.05–1	3(0.7–13)	1(0.7–7)	0.7(0.7–0.8)	1.29	0.2945

Using the ANOVA test for statistically difference in mean physical and chemical water properties with respect to water sources where the water samples were collected, apparent color, temperature, P^H^, dissolved oxygen and phosphorus were not statistically significant. Turbidity (F-Ratio-16.72), electrical conductivity (F-Ratio-9.14), ammonia (F-Ratio-39.44)), total suspended solids (F-Ratio-8.44) and chloride (F-Ratio-11.68) were statistically significant at P-Value of <0.001 as shown in Table 3.

**Table 3:** Median and interquartile range of physical chemical properties with respect to water source.

Water source	Borehole	Rain water	Tap water	Well	F-ratio	p-value
**Apparent Color (Median IQR)**	2(2–2)	10(0–71)	17(8–42)	34(20–114)	2.70	0.0613
**Temperature**	25(25–25)	23(21–24)	24(23–25)	23(22–25)	0.21	0.8917
**PH**	4,2(4.2–4.2)	6(4–6)	4(4–5)	4(4–4)	1.59	0.2108
**Turbidity**	5(5–5)	2(2–3)	4(3–8)	23(18–29)	16.72	<0.001
**Electrical conductivity**	905(905–905)	39(15–89)	118(115–124)	137(95–307)	9.14	<0.001
**Dissolved oxygen DO**	3(3–3)	4(3–4)	4(1–4)	1(0.8–4)	1.15	0.3447
**Phosphorus**	0(0–0)	1(0.7–2)	0.8(0.6–1)	0.8(0.6–1.4)	0.25	0.8609
**Ammonia**	8(8–8)	0.05(0–0.4)	0.2(0.1–0.3)	0.4(0.2–2.2)	39.44	<0.001
**Total suspended Solids**	452(452–452)	19(7–45)	59(57–62)	59(55–64)	8.44	<0.001
**Chloride**	2.3(2.3–2.3)	1(1–1)	0.7(0.7–0.7)	9(3–13)	11.68	<0.001

### Feacal coliforms isolated from the selected drinking water sources

*Citrobater divergens and E*. *coli were* the organisms with the highest percentage among the isolates from the drinking water samples from the selected water sources, at 62.16% and 35.14%, respectively. The mean log CFU/ml is 5.37 with a 2.57 standard deviation and an interquartile range of 3.4 to 6.23, as shown in Table 4.

**Table 4:** Feacal coliforms isolated from the selected drinking water sources.

	Frequency	Percentage Responses	Percentage of isolates
***Citrobacter divergenes***	23	43.40	62.16
***Citrobacter fluendii***	1	1.89	2.7
***Esherichia coli***	13	24.53	35.14
***Enterobacter aerogenes***	3	5.66	8.11
***Enterobacter agglomerus***	2	3.77	5.41
***Enterobacter cloacae***	5	9.43	13.51
***Klebsiella spp***	3	5.66	8.11
**No growth**	1	1.89	2.70
***Proteus mirabilis***	1	1.89	2.70
***Proteus species***	1	1.89	2.70

Physical properties measured were apparent color, temperature, pH, turbidity, electrical conductivity dissolved oxygen. Of the measured parameters, the mean of values for dissolved oxygen, Total suspended solids, and chloride were within the recommended standard for drinking water irrespective of water source. The chemical properties measured were phosphate, ammonia, nitrate, total dissolved solids and chloride. The mean of values total dissolved solids and chloride were within the permissible values for drinking water with values for phosphate slightly higher that the permissible value. Apart from apparent color, the mean values for rainwater and tapwater were within recommended standards as shown in the Table 5.

**Table 5:** Mean and standard deviation of physical- chemical Parameters and feacal coliforms isolated from selected drinking water sources with respect to water source.

Source	Apparent Color(TCUs)	Temp (°c)	P^H^	Turb (NTUs)	Ec(μs/cm	DO (mg/l)	PO_4_ (mg/l)	NH_4_ (mg/l)	TSS (mg/l)	Chl (mg/l	logCFU/ml)
**Borehole**	**2**	**24.7**	**4.65**	**4.2**	**905** *	**2.9**	**0**	**8.2** *	**452**	**2.3**	**5.1**
**Rainwater**	**27** * **(38.4)**	**22.6(1.8)**	**2.3(0.8)**	**5.4** * **(1.0)**	**47.3(37.7)**	**3.8(0.7)**	**1.4** * **(0.8)**	**0.2(0.2)**	**23.5(19.1)**	**1.3(0.1)**	**4.1(1.8)**
**Tap**	**26** * **.2(23.7)**	**23.7(2.4)**	**5.6(3.8)**	**4.4(0.9)**	**117.3(17.1)**	**3.2(1.4)**	**1.3** * **(1.9)**	**0.2(0.2)**	**62.2(21.9)**	**0.6(0.2)**	**5.4(2.6)**
**Well**	**72** * **(76.1)**	**23.1(4.7)**	**24.2** * **(3.0)**	**4.2(0.9)**	**285(321.1)**	**2.3(1.9)**	**1.8** * **(2.9)**	**1.1(1.4)**	**140.7(161.1)**	**8.8(7.0)**	**5.7(3.0)**
**Standards**	**15**	**25**	**8.5**	**5**	**300**	**5**	**1**	**2**	**500**	**250**	

*Mean of Values higher than permissible level

The mean of values for water sources in Kaburangire were within the recommended permissible values for drinking water. The mean of values for apparent color were higher than the permissible values except for Kaburangire. The mean values for electrical conductivity, dissolved oxygen, ammonia and chloride were within the permissible level irrespective of the village as shown in Table 6.

**Table 6:** Mean and standard deviation of physical- chemical parameters and feacal coliforms isolated from selected drinking water sources with respect to selected villages in Mbarara city.

Village	Apparent Color(TCUs)	Temp (°c)	PH	Turb (NTUs)	Ec(μs/cm	DO (mg/l)	PO_4_ (mg/l)	NH_4_ (mg/l)	TSS (mg/l)	Chl (mg/l	logCFU/ml)
**Kaburangire**	**10.7(12.4)**	**21.9(3.5)**	**5.9(3.5)**	**4.2(0.3)**	**204.5(244.6)**	**3.4(0.9)**	**1.1** * **(0.3)**	**0.7(1.4)**	**102.1(122.4)**	**2.6(4.8)**	**4.7(2.4)**
**Katukuru**	**52.4** * **(26.0)**	**23.2(1.2)**	**8.6** * **(11.3)**	**3.6(0.2)**	**126.1(27.7)**	**0.9(0.04)**	**2.9** * **(3.4)**	**0.2(0.3)**	**82.1(42.4)**	**0.9(1.4)**	**8.4(3.6)**
**Katebe**	**73.5** * **(88.4)**	**21.2(4.3)**	**21.6** * **(19.1)**	**3.7(0.4)**	**120.4(94.7)**	**0.8(0.06)**	**2.7** * **(3.8)**	**0.9(1.3)**	**57.4(44.3)**	**7.0(9.0)**	**6.0(3.1)**
**Lugazi**	**37** * **(64.7)**	**24.3(2.5)**	**11.7(11.7)**	**4.4(0.3)**	**288(391.5)**	**3.7(0.2)**	**0.5(0.3)**	**0.1(0.1)**	**141.1(197.4)**	**1.7(1.8)**	**4.8(0.5)**
**Nyarubanga**	**35(34.2)**	**25.8(2.2)**	**6.7(6.8)**	**5.9** * **(0.6)**	**110.6(43.4)**	**4.4(0.7)**	**0.9(0.5)**	**0.4(0.2)**	**55.3(21.7)**	**3.7(5.3)**	**4.2(1.9)**
**Rubiri**	**27.3** * **(18.8)**	**23.7(1.5)**	**9.5** * **(4.3)**	**4.1(0.1)**	**247.6(322.1)**	**3.8(0.5)**	**0.6(0.4)**	**1.6(3.3)**	**123.7(160.9)**	**1.0(0.7)**	**4.7(1.1)**
**Standard**	**15**	**25**	**8.5**	**5**	**300**	**5**	**1**	**2**	**500**	**250**	

*Mean of Values higher than permissible level

Apparent color, P^H^ and electrical conductivity, ammonia, total dissolved solids and chloride yielded four principal components with Eigenvalues close to one as shown in Table 7. They account for 78% of the total variance. Principal component 1(PC1) accounts for 25.80% of the total variance and exhibits a high negative loading (-0.44) with no significant positive loadings. PC2 and PC3 account for 23.17% and 19.50% of the total variance respectively. Thus exhibit significant positive loadings due to high apparent color, electrical conductivity, ammonia, total dissolved solids and chloride. PC4 accounts for 9.92% with a positive high loading of _P_^H^.

**Table 7:** Principal component loadings parameters of drinking water from selected drinking water sources.

Parameter	Coefficients of PC1	Coefficients of PC2	Coefficients of PC3	Coefficients of PC4
Apparent Color	0.07483	-0.0596	**0.54863**	-0.03373
Temperature	-0.35812	0.23926	0.21006	0.16877
P^H^	-0.36948	0.14376	-0.14709	**0.57004**
Turbidity	0.25	-0.04375	0.43179	0.3828
Electrical conductivity	0.29178	**0.47937**	-0.21719	0.02736
Dissolved oxygen(DO)	-0.4386	0.15625	-0.16361	0.30722
Phosphorus	0.30759	-0.30773	-0.28489	0.38528
Ammonia	0.23908	**0.46296**	0.05804	-0.04015
Total Dissolved Solids	0.2878	**0.48525**	-0.21608	0.02521
Chloride	0.15232	0.1952	**0.45273**	0.32784
Feacal Coliform	0.36309	-0.28249	-0.19149	0.38157
Eigenvalues	2.83848	2.54884	2.14487	1.09089
% of Variance	25.80	23.17	19.50	9.92
Cumulative %	25.80	48.98	68.47	78.39

Highlighted values in bold show values that account for variance in PC1, PC2, PC3 and PC4

**Fig 5:**
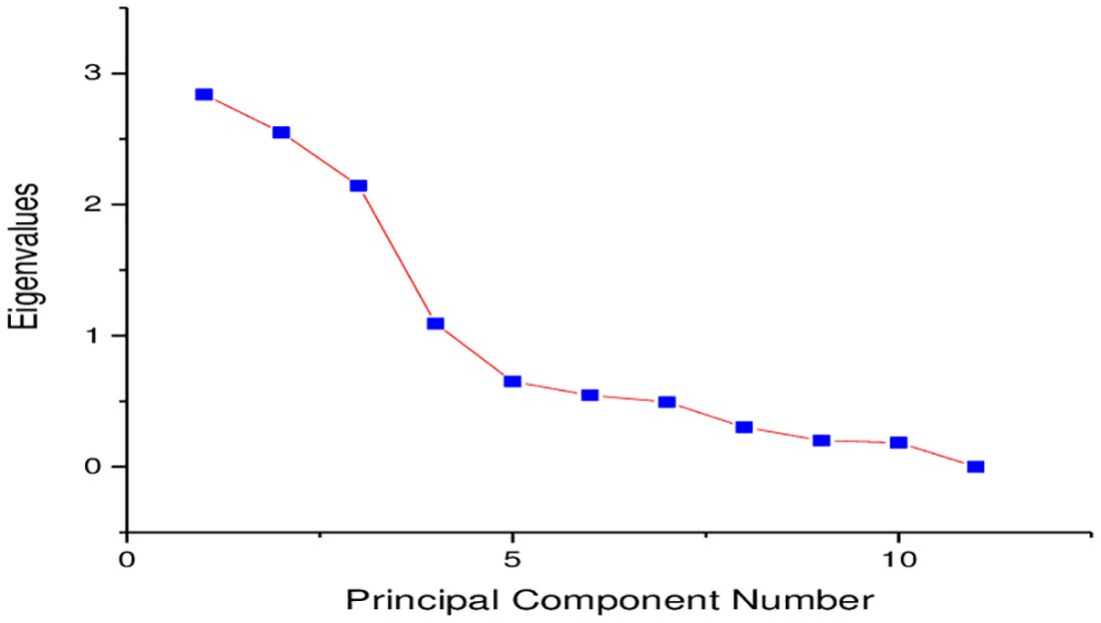
A scree plot of the parameters of drinking water from selected drinking water sources.

The Eigenvalues in Fig 5 start to level at Eigenvalue 0. 5 and PC5. The four principal components were preserved and account for 78% of the variance of the dataset.

**Fig 6:**
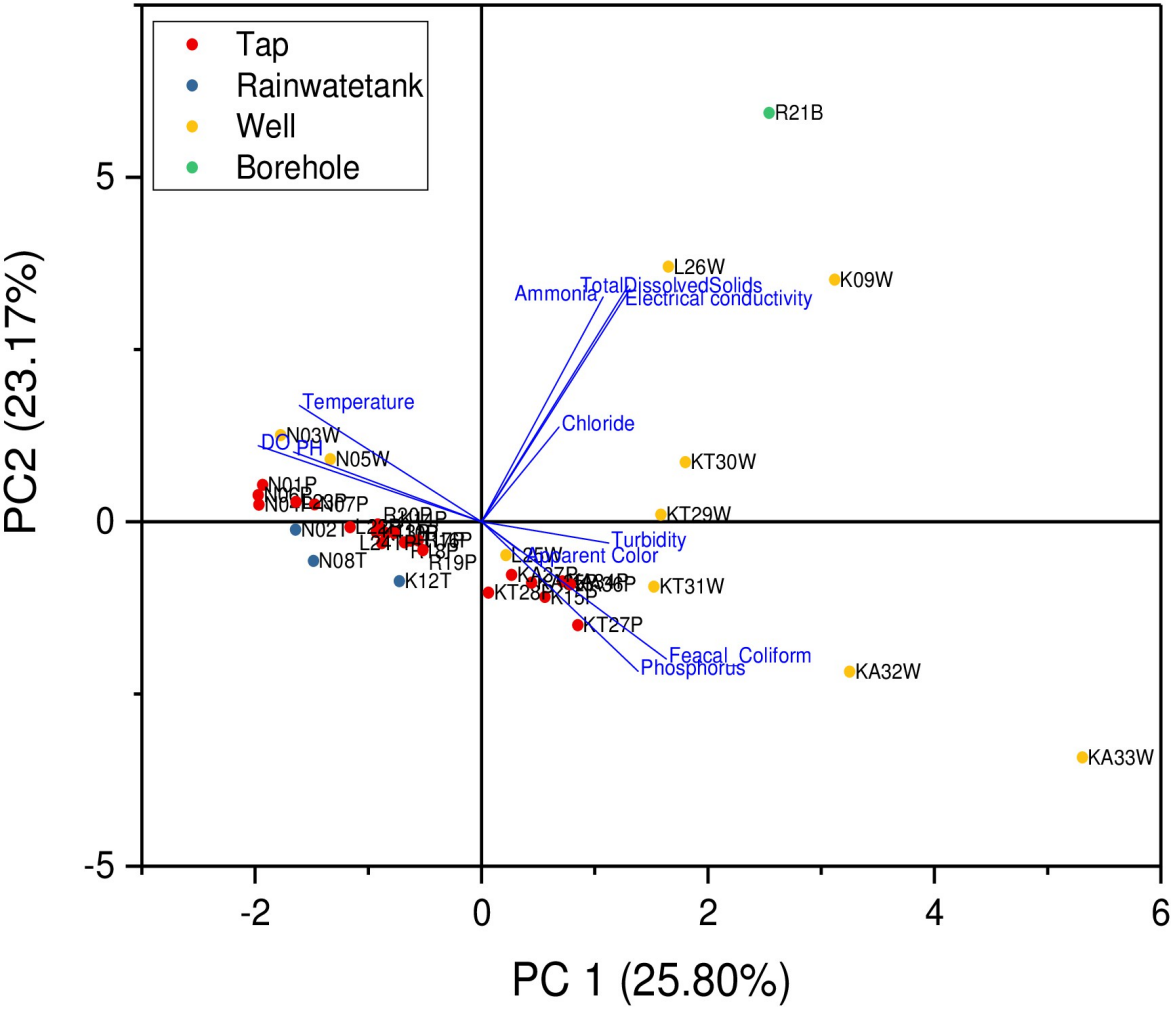
A biplot of principal component 1 and Principal component 2.

Turbidity, apparent color, phosphates and feacal coliform showed positive correlation with principal component 1. Ammonia, total dissolved solids and electrical conductivity showed a strong positive correlation with Principal component 2. Temperatures, dissolved oxygen and P^H^ showed a strong negative correlation with principal component 2 as shown in Fig 6.

**Fig 7:**
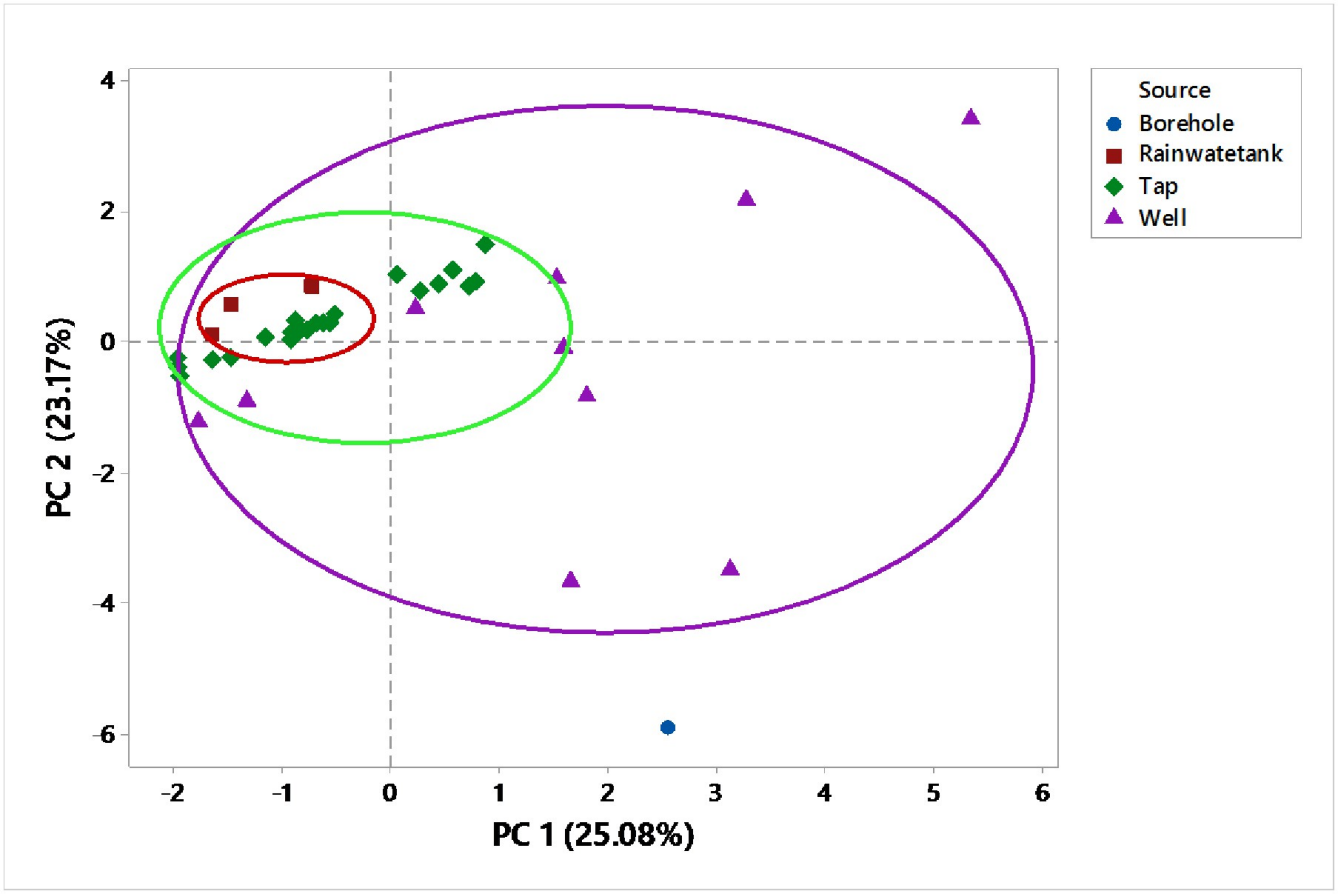
PCA for source apportionment.

Well, tap and borehole water accounts for the highest positive correlation in PC1 and PC 2 (Fig 7).

### Piper trilinear diagram for geochemical control for drinking water contamination

**Fig 8:**
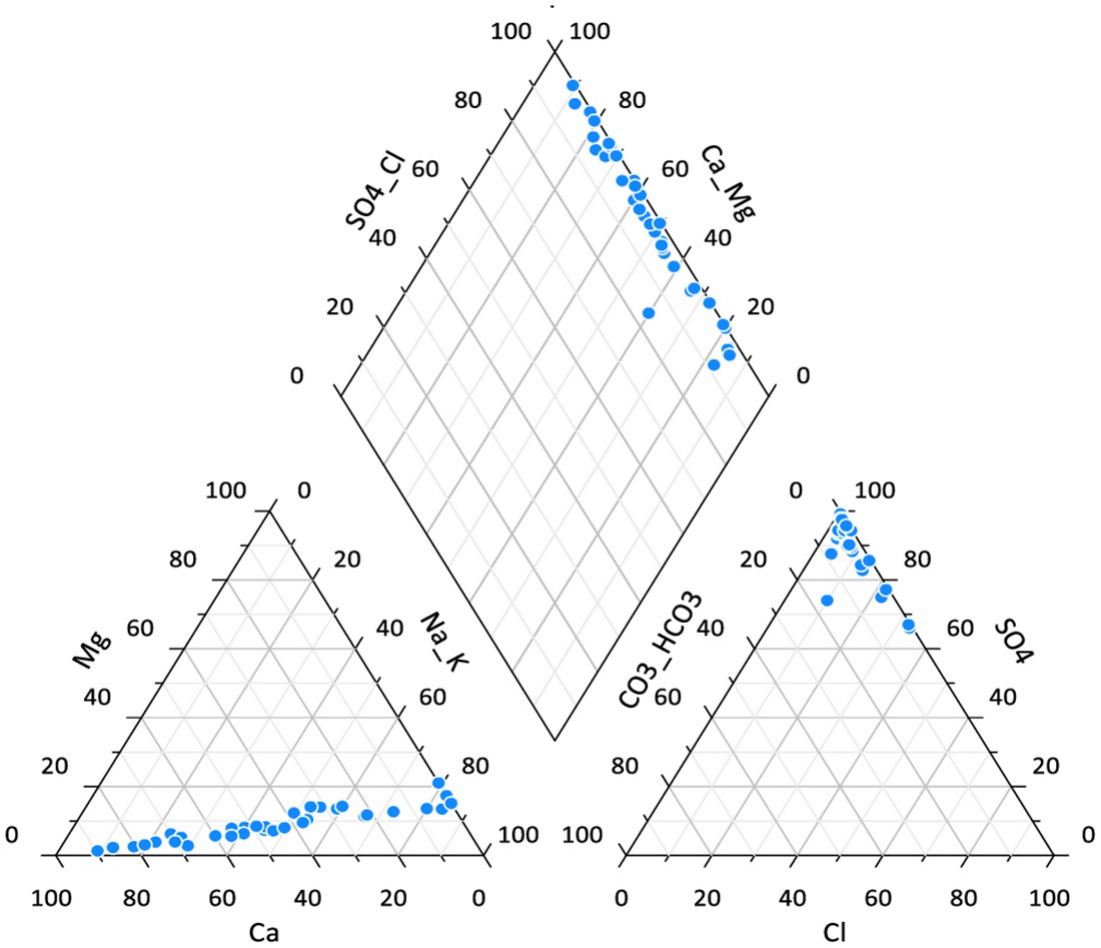
Piper trilinear diagram.

The higher density of Ca^2+-^_Mg_^2+^Cl^-^ is inclined toward the cation side of the triangle and So_4_ towards the anion side of the triangle. The drinking water shown by the central diamond plot Ca^2+^cl-type and Na ^+^cl-The drinking water is a mixed sulphate type (Calcium-Sodium sulphate) as shown in Fig 8.

### Cluster analysis of physical chemical and bacteriological parameters

**Fig 9:**
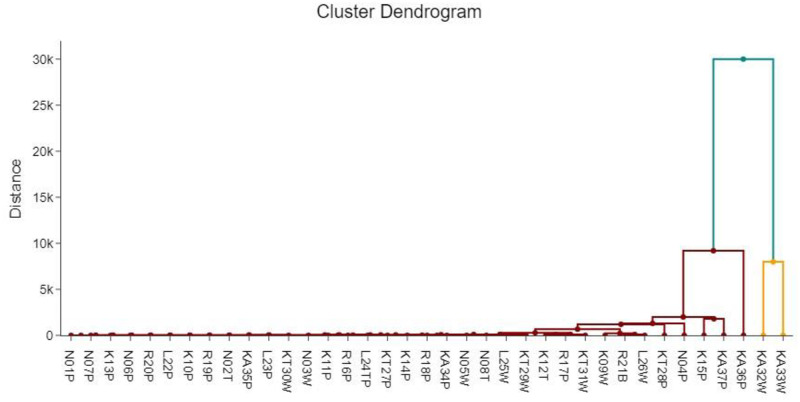
Cluster dendrogram of physical chemical and bacteriological parameters.

Two clusters were obtained. Cluster 1(n = 23), Cluster 2(n = 14). Cluster 1 contributes 25.81% and cluster 2 48.98%of the total variance as shown in Fig 9. The concentrations of the parameters in C1 and C2 are shown in Table 8.

**Table 8:** Concentrations of the physical chemical parameters in cluster 1 and cluster 2.

Cluster	Row	Color	Temp	PH	Turb	EC	DO	PO_4_	NH_4_	TSS	Chl
1	22	28	24.43	4.86	4.1	117.5	3.88	1.03	0.24	58.5	0.73
2	36	36	24.52	3.59	4.3	111	0.86	1.23	0.75	55	0.05

### Water quality index of selected drinking water sources

Of the 37 sampled water sources, Twelve water sources (32.4%) had water that was unfit for human consumption that was unfit for human consumption (Grade E), five sources (13.5%) had water that had a very poor index (Grade D), nine (24.3%) had water of poor index (Grade C), eight (21.6%) had water of good water index (Grade B) and only three (8.1%) had water of excellent water quality index (Grade A). As shown in Table 9.

**Table 9:** Water quality index and quality status of the selected drinking water sources.

					Physical Properties					Chemical Properties				
Code	Source	Color (TCU)	Temp °C	pH	Turb (NTU)	EC (μs/cm)	DO (mg/l)	PO_4_ (mg/l)	NH_4_ (mg/l)	TSS (mg/l)	Chloride (mg/l)	CFU/ML	WQI	Status
**N01P**	**Tap**	**12**	**25.76**	**5.9**	**3.7**	**124**	**4.41**	**0.5**	**0.71**	**62**	**0.68**	**17**	**37.806**	**Good**
**N02T**	**Rain water tank**	**71**	**24.13**	**6.3**	**3.2**	**89**	**4.5**	**2.2**	**0.45**	**45**	**1.4**	**24**	**119.220**	**Unfit**
**N03W**	**Well**	**37**	**28.62**	**5.8**	**155**	**157**	**5.39**	**0.6**	**0.47**	**79**	**12.3**	**20x10**	**339.642**	**Unfit**
**N04P**	**Tap**	**14**	**24.59**	**6.3**	**3.6**	**121**	**4.78**	**0.8**	**0.26**	**61**	**0.7**	**30x10** ^**2**^	**56.860**	**Poor**
**N05W**	**WELL**	**103**	**29.42**	**5.4**	**193.5**	**117**	**4.76**	**1**	**0.66**	**59**	**12.24**	**68**	**433.523**	**Unfit**
**N06P**	**Tap**	**16**	**25.17**	**6.8**	**3.3**	**138**	**3.72**	**0.8**	**0.21**	**69**	**0.66**	**0**	**106.936**	**Unfit**
**N07P**	**Tap**	**17**	**25.28**	**4.9**	**2.9**	**126**	**4.51**	**0.8**	**0.55**	**63**	**0.73**	**19**	**53.118**	**Poor**
**N08T**	**Rain water tank**	**10**	**23.12**	**5.5**	**2**	**15**	**3.14**	**0.7**	**0**	**7**	**1.28**	**20**	**36.389**	**Good**
**K09W**	**Well**	**29**	**19.62**	**4.2**	**3.7**	**754**	**1.28**	**1.4**	**3.85**	**377**	**13.4**	**40**	**121.451**	**Unfit**
**K10P**	**Tap**	**0**	**24.02**	**4.7**	**3.6**	**142**	**3.43**	**1.5**	**0.12**	**71**	**0.71**	**20**	**83.359**	**Very poor**
**K11P**	**Tap**	**0**	**25.08**	**4.4**	**10.8**	**125**	**3.81**	**1.5**	**0.17**	**62**	**0.67**	**21x10**	**96.482**	**Very poor**
**K12T**	**Rain water tank**	**0**	**20.64**	**4.3**	**1.9**	**39**	**3.69**	**1.2**	**0.05**	**19**	**1.3**	**50x10**	**62.813**	**Poor**
**K13P**	**Tap**	**8**	**24.02**	**4.1**	**3.7**	**128**	**3.95**	**0.8**	**0.08**	**64**	**0.67**	**4**	**45.743**	**Good**
**K14P**	**Tap**	**26**	**24.62**	**4**	**9.4**	**123**	**3.85**	**0.8**	**0.19**	**61**	**0.68**	**119**	**58.160**	**Poor**
**K15P**	**Tap**	**12**	**15.56**	**3.9**	**8.1**	**123**	**3.74**	**0.9**	**0.13**	**61**	**0.72**	**68x10** ^**2**^	**194.241**	**Unfit**
**R16P**	**Tap**	**24**	**22.94**	**4.3**	**17.7**	**117**	**4.08**	**0.8**	**0.25**	**58**	**0.67**	**204**	**75.071**	**Very poor**
**R17P**	**Tap**	**16**	**22.64**	**4.1**	**8.3**	**116**	**4.04**	**0.8**	**0.34**	**58**	**0.73**	**49x10**	**61.156**	**Poor**
**R18P**	**Tap**	**42**	**24.17**	**3.9**	**8.4**	**116**	**3.8**	**1**	**0.15**	**58**	**0.66**	**120**	**65.447**	**Poor**
**R19P**	**Tap**	**55**	**21.84**	**3.9**	**8.6**	**114**	**4.25**	**0.8**	**0.22**	**57**	**0.73**	**32**	**58.688**	**Poor**
**R20P**	**Tap**	**25**	**25.96**	**3.9**	**9.2**	**118**	**3.61**	**0.4**	**0.19**	**59**	**0.75**	**3x10**	**39.915**	**Good**
**R21B**	**Borehole**	**2**	**24.7**	**4.2**	**4.7**	**905**	**2.87**	**0**	**8.24**	**452**	**2.3**	**168**	**110.262**	**Unfit**
**L22P**	**Tap**	**28**	**24.43**	**4.9**	**4.1**	**118**	**3.88**	**1**	**0.24**	**59**	**0.73**	**0**	**69.071**	**Poor**
**L23P**	**Tap**	**1**	**28.1**	**4.5**	**3**	**122**	**3.63**	**0.2**	**0.07**	**59**	**0.72**	**63**	**14.908**	**Excellent**
**L24P**	**Tap**	**0**	**22.73**	**4.1**	**3**	**118**	**3.98**	**0.6**	**0.04**	**47**	**0.9**	**184**	**34.107**	**Good**
**L25W**	**Well**	**151**	**21.56**	**4.1**	**28.1**	**95**	**3.64**	**0.3**	**0.26**	**48**	**4.98**	**182**	**78.289**	**Very poor**
**L26W**	**Well**	**5**	**24.66**	**4.3**	**11.1**	**988**	**3.47**	**0.4**	**0.05**	**494**	**1.16**	**113**	**40.749**	**Good**
**KT27P**	**Tap**	**57**	**22.32**	**4.4**	**1.6**	**112**	**0.91**	**0.3**	**0.07**	**57**	**0.7**	**17x10**	**18.613**	**Excellent**
**KT28P**	**Tap**	**7**	**21.91**	**3.8**	**2**	**47**	**0.88**	**0.6**	**0.05**	**24**	**0.67**	**17x10** ^**2**^	**65.354**	**Poor**
**KT29W**	**Well**	**233**	**23.6**	**3.4**	**25**	**87**	**0.77**	**0**	**2.24**	**44**	**12.53**	**13x10**	**76.176**	**Very poor**
**KT30W**	**Well**	**30**	**23.12**	**3.6**	**25**	**63**	**0.77**	**0**	**2.9**	**44**	**22.94**	**4**	**802.640**	**Unfit**
**KT31W**	**Well**	**114**	**24.04**	**3.6**	**31.7**	**107**	**0.79**	**1.1**	**0.21**	**32**	**4.43**	**51x10**	**116.588**	**Unfit**
**KA32W**	**Well**	**20**	**23.25**	**3.8**	**51.7**	**176**	**0.81**	**3.8**	**0.32**	**88**	**3.21**	**54x10** ^**3**^	**396.109**	**Unfit**
**KA33W**	**Well**	**0**	**12.74**	**3.4**	**51.7**	**307**	**0.87**	**9.3**	**0.05**	**145**	**0.87**	**46x10** ^**3**^	**145.310**	**Unfit**
**KA34P**	**Tap**	**53**	**23.03**	**2.2**	**3.8**	**116**	**0.92**	**0.2**	**0.04**	**58**	**0.06**	**12x10**	**17.661**	**Excellent**
**KA35P**	**Tap**	**87**	**21.56**	**12.5**	**5.4**	**114**	**0.93**	**0.4**	**0.02**	**57**	**0.07**	**9**	**32.0383**	**Good**
**KA36P**	**Tap**	**36**	**24.52**	**3.6**	**2**	**111**	**0.86**	**1.2**	**0.75**	**57**	**0.07**	**16x10** ^**3**^	**32.038**	**Good**
**KA37P**	**Tap**	**66**	**24.37**	**3.7**	**4.3**	**115**	**0.85**	**0.7**	**0.04**	**55**	**0.05**	**50x10** ^**2**^	**385.945**	**Unfit**
**Standards(WHO, US EAS 12)**		**15**	**25**	**8.5**	**5**	**300**	**5**	**1**	**2**	**500**	**250**	**500**		

## Discussion

The water samples were tested for physical properties (apparent color, temperature, pH, turbidity, and electrical conductivity), chemical properties (dissolved oxygen, phosphates, ammonia, and total suspended solids), and fecal coliforms. The values for apparent color and phosphates were higher than the permissible values. Color changes are a result of presence of dissolved colloidal substances and materials in water. Presence of humic acids, fulvic acids, metallic ions, suspended matter, phytoplankton, industrial effluents, algal flora, organic matter and iron in water lead to its change in color, taste and odor [24]. Phosphorus is key to the eutrophication of aquatic ecosystems, leading to increased nutrient concentration and consequently an increase in productivity. Excessive levels of phosphorus lead to algae blooms, anoxic conditions, water acidification, which leads to dead zones, toxin production, and health issues [25]. Phosphorus accumulation, results in a high risk of phosphorus pollution due to high multiple vegetable cropping indexes and excessive fertilizer input [26]. The higher concentration of PO_4_ resulted from precipitation and evaporation. The values for P^H^, electrical conductivity, dissolved oxygen, ammonia, total suspended solids, and chloride were within the permissible levels according to the recommended guidelines for drinking water in Uganda and the World Health Organization. The value for turbidity were close to the maximum of permissible value. Turbidity is a measure of water clarity and is a major determinant of water condition and productivity. The more turbid the drinking water appears, the higher the measured turbidity values [27]. Turbidity of drinking water is caused by presence of suspended particles that hinders the conduction of light through water [28]. The values for turbidity from this study are lower than the permissible limits for drinking water by the National water and sewerage corporation and Uganda standards for drinking water of ≤25 NTU. This is however higher than the results of a study by Edokpayi and co-authors, where the mean values obtained for different seasons were higher than the SANS and WHO permissible limits of ≤1 NTU for domestic water use, and the average turbidity values varied significantly for both the wet and dry seasons. This difference can be traced back to the differences in standards of grading in different environments [29]. Dissolved oxygen is a measure of the degree of pollution by organic matter coupled with the destruction of organic substances and tests water source purification. It determines the dynamics of biodata and helps to regulate several metabolic processes in drinking water. Dissolved oxygen is one of the most important factors in the existence of aquatic life. Dissolved oxygen concentration has a significant effect on groundwater quality by regulating the valence state of trace metals and by constraining the bacterial metabolism of dissolved organic species [30]. Dissolved oxygen in open dug wells was lower compared to piped tap water and rain harvest tank water, but generally, the values fall within the permissible ranges for dissolved oxygen in drinking water. A study on Spatial and temporal dynamics of water quality in aquatic ecosystems in rivers in Malawi showed that DO were at an alarming level due to non-point source pollution [31]. A study in the divisions of Nyamitanga, Kamukuzi, and Kakoba divisions of Mbarara city found that the mean DO values were between 4.84 and 12.86 mg/l and the results that were almost similar to the results of this study [32]. P^H^ Value in this study lies within the permissible values of ≤ 8.5. Similarly, a study on groundwater sources, surface runoff, wastewater, and surface water from designated streams in Lake Victoria basin, Uganda, found that the shallow groundwater was acidic with pH values below 6.5 [33]. It should be noted that pH is an important characteristic of water and a basic water quality indicator. Small changes in its level disorganize the quality of the water. pH influences the availability of micronutrients and trace metals [34]. Electrical conductivity is a measure of the ability of water to conduct electric current. This ability to conduct current depends on the concentration of ions, temperature, and ionic mobility. Electrical conductivity measures the dissolved solids in water bodies, hence the variations in Electrical conductivity depends on the fluctuations in salinity and total dissolved solids. The electrical conductivity is directly proportional to the dissolved matter. Electrical conductivity for all sources lies within the recommended standards of ≤2500 (μs/cm) except for boreholes. The results of this study are similar to the results of the study by Sitotaw and others on the seasonal dynamics in bacteriological and physicochemical water quality of the southern gulf of Lake Tana, where the values of electrical conductivity fell within permissible ranges during the dry and wet seasons of the year [35]. The difference in electrical conductivity of borehole water can be attributed to the fact that the safety of borehole water is subject to the condition of the infrastructure (pump and distribution system) provided and the site of the borehole [36]. Ammonia contains nitrogen and hydrogen. It is one of the most important pollutants since it can be toxic to aquatic life, leading to lower production, growth, and death. The levels of ammonia in this study were within permissible values for drinking water. These values are within the recommended standard for drinking water in Uganda, except for boreholes. Ammonia is ubiquitous in nature and in surface water [37]. Chloride is required for normal cell functions in plant and animal life, though it is required in small quantities. Elevated levels of chloride are an indicator of water pollution. This affects aquatic life as it interferes with osmoregulation, a biological process by which organisms maintain their proper concentrations of salt and other solutes in body fluids. Chloride is the most dominant anion in water. The values for chloride in this study were within the permissible levels for drinking water. Generally, an analysis of the physical and chemical properties of drinking and domestic water sources in cholera-prone communities in Uganda found that all sites (100%) had mean water turbidity values greater than the WHO drinking water recommended standards and a temperature above 17°C. It should be noted that 27% of the lake sites and 2/5 of the ponds had pH and dissolved oxygen, respectively, outside the WHO recommended range of 6.5–8.5 for pH and less than 5 mg/l for dissolved oxygen [38].

The piper trilinear revealed that the dominant water type of the area were Caso_4_and Naso_4_ type. Gibbs plot represents majorly precipitation and miner evaporation dominance. The geochemical process of precipitation of Mbarara city are influenced by chemical characteristics and hence responsible for the variation in drinking water. Similar to our study; Caso_4_and Naso_4_ type was the dominant water type and precipitation are influenced the chemistry of water in urban areas of Kuwait though it was combined with dissolution that is not the case in this study area [39]. PCA for source apportionment showed that well, tap and borehole water account for the highest variations in the quality of drinking water. The Cluster analysis supported the PCA analysis. The Cl^-^ and So4 contamination resulted from anthropogenic sources like waste, Agriculture, fertilizers and atmospheric sources [40].

*Citrobater divergens and E*. *coli* were the highly isolated fecal coliforms. Safe drinking water is required for all usual domestic purposes like drinking, food preparation, and personal hygiene. Safe drinking water should not represent any significant risk to health over a lifetime of consumption [2]. A breakdown in water supply safety (source, treatment, and distribution) and available water management policies may lead to large-scale contamination and potentially detectable disease outbreaks [41]. Diseases related to the contamination of drinking water constitute a major burden on human health. The people at greatest risk of waterborne disease are infants and young children, people who are debilitated, and the elderly, especially when living in compromised sanitary environments. Drinking water generally contains diverse microorganisms whose growth and interactions are regulated by the type and concentration of available organic and inorganic nutrients, the type and concentration of residual disinfectant, environmental conditions such as temperature and water bulk, sediment and biofilm, and global climate change that results in changes in ambient temperature, heavy rainfall, drought, and flooding [42]. There was a strong relationship between bacterial contamination and temperature [43]. Similarly, a study by [44] suggests that droughts and heavy rainfall and the significant effects of initial soil moisture conditions on water shed affect water quantity and quality. Ideally, drinking water should be available to consumers when total viable counts are at 22 ºC in ml, total viable counts at 37 ºC in ml are at 100 and 50, respectively, and total coli in 100 ml and *E*. *coli* in 100 ml are absent as per Uganda standard for drinking water. Results from this study indicate that most sources did not meet the recommended standard guidelines, similar to the study conducted in Kisoro, where most drinking water sources were found to have coliform counts above the recommended national and international guidelines [45]. Microbiological stability of drinking water is key to ensuring that consumers access safe and stable drinking water of the same microbial quality at the end-user point as was supplied at the treatment facility [46]. Due to several factors like the development of opportunistic pathogens, deterioration of taste, odor, color, and biocorrosion of pipes during distribution in water mains, individual premise plumbing, leakages in the distribution lines due to human activities like cultivation, construction, and road construction, and routine road maintenance works [47].

The majority of the community members in Mbarara City drew their drinking water from piped water supplied by the National water and Sewerage Corporation, compared to open dug wells, rainwater harvesting tanks, and boreholes. A similar study conducted in Bushenyi Ishaka municipality found that households in more urban (as compared to rural) cells were more likely to use improved water sources (including piped water on-premises), make regular payments for water, rely on shared sanitation facilities, and make use of manual sludge emptying services [48]. This study found that twelve (12) sources had water that was unfit for human consumption, five (5) sources had water that had a very poor index, nine (9) had water of poor index, eight (8) had water of good water index, and only three (3) had water of excellent water quality index. Though the water samples collected from some taps were poor and unfit for human consumption, at least there were taps that had excellent water quality status as per the water quality index per individual source compared to open-dug wells, boreholes, and rain harvest tanks. This suggests contamination along the distribution and outlet of the water. Ranking from highest to lowest microbiological quality of water sources follows as boreholes, roof water harvesting, and open dung wells [49].

## Conclusion and recommendation

The values for apparent color and phosphate were higher than the permissible level as set by the World Health Organization and the Uganda guidelines for drinking water quality.

The isolated organisms were *Klebsiella spp*. (8.11%), *Citrobacter divergens* (62.16%), *Citrobacter fluendii* (2.7%), *E*. *coli* (35.14%), *Enterobacter aerogenes* (8.11%), *Enterobacter agglomerus* (5.4%), *Proteus spp*. (2.7%), *Enterobacter cloacae* (13.5%), and *Proteus mirabilis* (2.7%).

Twelve water sources (32.4%) had water that was unfit for human consumption (Grade E), five sources (13.5%) had water that had a very poor index (Grade D), nine (24.3%) had water of poor index (Grade C), eight (21.6%) had water of good water index (Grade B), and only three (8.1%) had water of excellent water quality index (Grade A).

The piper trilinear revealed that the dominant water type of the area were Mgso_4_ and Caso_4_ type. Gibbs plot represents precipitation dominance. PCA for source apportionment showed that well, tap and borehole water account for the highest variations in the quality of drinking water.

These results suggest that drinking water from sources in Mbarara city not suitable for direct human consumption without treatment. We recommend necessary improvements in water treatment, distribution, and maintenance of all the available water sources in Mbarara city, south-western Uganda.

### Implications to policy

Our findings highlight information on the physical, chemical parameters and fecal coliform and water quality index of drinking water from selected drinking water sources in Mbarara city. The findings in our study therefore show that water from these sources may pose severe health risks to consumers and is unsuitable for direct human consumption without treatment. The water management system needs enhancement to include testing, monitoring, and routine surveillance of all the water sources in use by the community, not just the gazetted ones, as per the policy of the Ministry of Water, Lands, and Environment since the community obtains water for drinking from all the available sources other than the gazetted ones concurrently. This can be helpful in providing and maintaining a safe and quality drinking water supply for the community. This should entail routine testing, sanitary inspection, and giving feedback in simple language that can be understood by the end-users of drinking water in the community.

## Supporting information

S1 Dataset (XLS)

## Abbreviations

APHA: American Public Health Association
CDC: Centers for Disease Control and Prevention
EAS: East African Standards
EC: Electrical conductivity
MPN: Most probable number SANS South African National Standards
NEMA: National Environment Management Authority
NWSC: National Water and Sewerage Cooperation
TDS: Total Dissolved Solids
UBOS: Uganda Bureau of Statistics
WASH: Water, sanitation, and hygiene
WHO: World Health Organization
